# Structural characterization and inhibition of carbonic anhydrase from *Candida parapsilosis*

**DOI:** 10.1016/j.yjsbx.2025.100140

**Published:** 2025-11-12

**Authors:** Jiří Dostál, Zdeňka Uhrová, Magdalena Škrlová, Stanislav Macháček, Kamila Clarová, Martin Lepšík, Ondřej Bulvas, Milan Vrábel, Olga Heidingsfeld, Iva Pichová

**Affiliations:** a Institute of Organic Chemistry and Biochemistry of the Czech Academy of Sciences, Flemingovo náměstí 2, 166 10 Prague, Czech Republic; b Department of Biochemistry, Faculty of Science, Charles University in Prague, Hlavova 2030, 128 43 Prague, Czech Republic

**Keywords:** β-class carbonic anhydrase, *Candida parapsilosis*, *CpNCE103p*, X-ray crystallography, Acetazolamide

## Abstract

Fungal carbonic anhydrases (CAs) are metalloenzymes that catalyze the reversible hydration of carbon dioxide and play an essential role in fungal adaptation to environments with fluctuating CO_2_ concentrations. The opportunistic pathogen *Candida parapsilosis* expresses a single β-class CA, CpNce103p, which is structurally distinct from human α-class CAs and therefore may represent a promising antifungal target. Here, we report the biochemical and structural characterization of CpNce103p, including its crystal structure in complex with the classical CA inhibitor acetazolamide. Mass photometry and X-ray crystallography revealed that CpNce103p forms a stable homotetramer, similar to its homolog CaNce103p from *C. albicans*. Inhibition constants (Ki) were measured for a panel of 16 sulfonamide derivatives. One compound attained single-digit micromolar inhibition of CpNce103p, similar to the Ki of acetazolamide. Docking suggested the compound’s binding mode, featuring zinc coordination and accommodation within the tight cavity. Our findings provide a structural basis for rational inhibitor design targeting fungal β-CAs and support CpNce103p as a viable target for the development of antifungal agents with selective activity.

## Highlights

• First crystal structure of a complex of beta carbonic anhydrase from Candida parapsilosis with acetazolamide (AZM) inhibitor.
• Novel potent sulfonamide inhibitor identified (Ki ∼ 2 μM).
• Docking predicts inhibitor binding modes consistent with AZM co-crystal pose.
• Zn^2+^ coordination and active-site steric constraints govern inhibitor binding.
• Zn^2+^ anchoring and narrow active-site channel provide basis for inhibitor design.

## Graphical abstract

**Figure:**
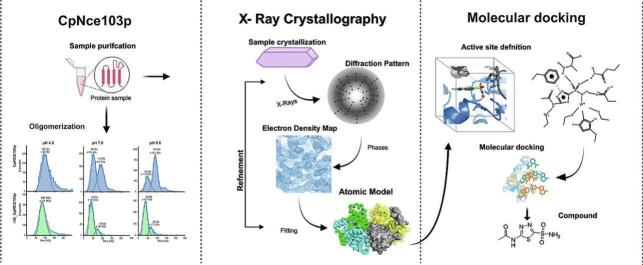

## Introduction

Reversible hydration of carbon dioxide to bicarbonate proceeds spontaneously in nature. However, regulating the balance between CO_2_ and HCO_3_^−^ is so important that specialized enzymes catalyzing the reversible reaction CO_2_ + H_2_O ↔ HCO_3_^−^ + H^+^ have evolved several times independently. These metalloenzymes, called carbonic anhydrases (CAs), exist in all kingdoms of life (Capasso and Anhydrases, 2023). They are involved in numerous physiological processes, CO_2_ and HCO_3_^−^ sensing and transport, as well as maintaining acid–base balance. Their convergent evolution is reflected in their structural dissimilarity. The extant CAs are classified into eight genetically distinct families: ɑ, β, ɣ, δ, ζ, ƞ, θ, and ι (Supuran, 2023). While their overall architectures differ, their active sites are similar, typically consisting of a divalent metal ion coordinated by three amino acid residues along with either a molecule of water or a hydroxyl. Zinc is the most common ion, but CAs containing cobalt, iron, cadmium, or manganese have also been identified and studied (Kim et al., 2020). Metal-hydroxyl species in the active site are essential for CA activity, and their formation – through proton release from the catalytic water molecule to the environment – is the rate-limiting step of CO_2_–HCO_3_ interconversion (Supuran, 2016). Substrate turnover rates are close to diffusion-controlled limits, placing CAs among the fastest enzymes in nature (Aggarwal et al., 2015). This reflects the physiological importance of the rapid adaptation of cells to changes in CO_2_ concentrations.

CA inhibitors are used to treat a diverse set of conditions, including seizure disorders, altitude sickness, and glaucoma. They are also components in certain diuretic drugs. The general principles of CA inhibition include binding the zinc atom in the active site, anchoring the catalytic water molecule, and blocking entrance to the active site (Supuran, 2016). Classical inhibitors based on zinc binding comprise the sulfonamides and their derivatives, containing an SO_2_NH^−^ zinc-binding group. Carboxylates, hydroxamates, and dithiocarbamates can play a similar role (Supuran, 2016). By binding the zinc atom, these compounds displace the catalytic water. This water molecule can also be anchored – that is, bound by externally added compounds through hydrogen bonding. This anchoring prevents the water from acting as a nucleophile and from binding CO_2_. Polyamines, phenols, and sulfocoumarins inhibit CAs via this mechanism (Lomelino et al., 2016). Active site-occluding inhibitors are specific for particular CA molecules due to structural differences among CA families. Though this may confer an advantage by ensuring inhibitor selectivity, diversion from the general principles can also make inhibitor design more challenging. Coumarin-based molecules are one of the many classes of compounds developed as inhibitors of human CAs (Lomelino et al., 2016).

*Candida parapsilosis* is an opportunistic yeast pathogen that can colonize humans as a harmless commensal. However, in individuals with impaired immune responses or disturbed microbiome composition, it can cause infections ranging from mild superficial mycoses to life-threatening systemic diseases (Pappas et al., 2018). Although traditionally considered less virulent than *C. albicans*; *C. parapsilosis* has become an increasingly important cause of invasive candidiasis, ranking second or third among pathogenic *Candida* species depending on geographical region and clinical setting. It is particularly prevalent in neonatal intensive care units (Pammi et al., 2013, Toth et al., 2019). A key factor in its pathogenicity is the ability to adhere to extracellular matrix proteins and to form robust biofilms on both biotic and abiotic surfaces (Alves et al., 2024, Gomez-Molero et al., 2021, Kozik et al., 2015, Michalcova et al., 2024). These biofilms facilitate transmission within healthcare environments, whether via contaminated surfaces and indwelling medical devices such as catheters, or through the hands of healthcare personnel. The emergence of *C. parapsilosis* strains resistant to commonly used antifungal agents has been reported in recent years (Daneshnia et al., 2023). This rise in incidence and resistance underscores the urgent need to identify novel therapeutic targets and compounds capable of interfering with essential biological processes in *C. parapsilosis*.

Microbiota inhabiting both external and internal niches of mammalian bodies are exposed to extreme differences in CO_2_ concentrations. The skin and mucosa come in contact with atmospheric air containing 0.04 % CO_2_, whereas blood contains at least 5 % CO_2_. Pathogenic fungi are among the microorganisms that can cope with these changes. Thriving on both the skin and in the blood or internal organs of mammalian hosts would not be possible without carbonic anhydrases. Fungal and yeast species usually possess β- or γ-type CAs, unlike mammals, which express several isoforms of α-CAs (15 found in humans). The absence of β-type CAs from human hosts makes them potential drug targets (Akocak and Supuran, 2019).

The gene *NCE103*, which encodes CA in the pathogenic yeast *Candida albicans*, is essential when the yeast grows in atmospheric air. However, in mammalian blood, where CO_2_ concentrations are much higher, CA is not needed and *NCE103* is not transcribed (Klengel et al., 2005). Therefore, Nce103p inhibition would not alleviate systemic candidiasis. On the other hand, the high rate of hospital-acquired *Candida* infections highlights the need for novel disinfectants to decontaminate surfaces in healthcare facilities. In addition, inhibitors of fungal CAs could be efficient as a topical treatment for superficial candidiasis.

In *C. parapsilosis* as well as in *C. albicans*, Nce103p is localized on the cell surface and plays a role in CO_2_ sensing (Dostal et al., 2020, Martin et al., 2017). The solved structure of *C. albicans* carbonic anhydrase – CaNce103p – shows that the enzyme forms a homotetramer arranged as a dimer of dimers. The narrow-tunnel shape of the entrance to the CaNce103p active site must be considered in the design of potential inhibitors (Dostal et al., 2018). The aim of the present study was to structurally analyze the *C. parapsilosis* CA, CpNce103p, and test a panel of its potential inhibitors.

## Materials and methods

### Cloning and expression of CpNce103p

*CpNCE103* sequences used in this study can be found in GenBank (and in the *Candida* Genome Database) under accession number CCE40150.1 (**CPAR2_101880**). The DNA used for gene amplification was isolated from *C. parapsilosis* strain P69, obtained from the mycological collection of the Faculty of Medicine, Palacky University, Olomouc, Czech Republic. The CpNce103p coding sequence and its truncated version, Δ42_CpNce103p, were cloned using the primers listed in Table S1 and inserted into the *E. coli* expression vector pET-22b(+) – linearized with the NdeI and XhoI restriction enzymes – using the InFusion HD Cloning Kit (Clontech). All PCR-derived DNA segments were verified by sequencing.

For protein expression, *E. coli* BL21(DE3) transformants were grown in Luria–Bertani (LB) medium supplemented with 100 µg/mL ampicillin and 1 mM ZnSO_4_. Cultures were incubated at 37 °C with shaking (220 rpm) until mid-log phase (OD_600_ ≈ 0.6). Expression of CpNce103p was induced by adding isopropyl β-D-1-thiogalactopyranoside (IPTG) to a final concentration of 0.4–0.5 mM. After induction, the cultivation temperature was lowered to 20 °C and the shaking speed reduced to 180 rpm. Cells were then incubated under these conditions for 16–20 h to allow protein production. This reduced-temperature, overnight expression protocol increased the yield of soluble CpNce103p. Following expression, cells were harvested by centrifugation at 4000×*g* for 10 min, and the cell pellets were either used immediately or stored at –20 °C.

### Purification of CpNce103p

CpNce103p and Δ42_CpNce103p were recovered in the soluble fraction following cell lysis and initially purified by Ni^2+^-affinity chromatography using a HiTrap Ni column (GE Healthcare) pre-equilibrated with 10 mM Tris-HCl (pH 8.0) and 500 mM NaCl. Bound proteins were eluted with a linear imidazole gradient (0–0.5 M). Fractions containing CpNce103p (Δ42_CpNce103p) were identified by SDS-PAGE, pooled, and subjected to buffer exchange using a HiPrep 26/10 Desalting column (GE Healthcare) packed with Sephadex G-25 Fine. This step was used to remove imidazole and transfer the protein into the anion-exchange chromatography buffer, consisting of 10 mM Tris-HCl (pH 8.0), 2 mM TCEP, and 10 % (v/v) glycerol. As the final purification step, anion-exchange chromatography was then performed using a MonoQ column pre-equilibrated with the same buffer. CpNce103p (Δ42_CpNce103p) was eluted using a linear NaCl gradient (0–1 M). The purity and homogeneity of the protein was assessed by SDS-PAGE, and its identity was confirmed by N-terminal sequencing and MALDI-TOF mass spectrometry.

### Mass photometry analysis

To assess pH-dependent changes in the oligomeric state of CpNce103p and Δ42_CpNce103p, protein samples were diluted in measurement buffers across a pH range of 4.0–9.0. Buffers were prepared as follows: 150 mM NaCl and 20 mM NaH_2_PO_4_ (pH 4.0 and 5.0); 20 mM MES (pH 6.0); and 20 mM Tris-HCl (pH 7.0, 8.0, and 9.0). The final protein measurement concentration was 20 nM. Mass photometry experiments were performed at room temperature using a TwoMP automated mass photometer (Refeyn). The instrument was calibrated according to the manufacturer’s protocol using 10 nM bovine serum albumin (BSA) and immunoglobulin G (IgG) standards diluted in measurement buffer (150 mM NaCl, 20 mM Tris-HCl, pH 7.0). Focusing was performed using the droplet dilution method. Scattering movies were recorded for 60 s and analyzed using DiscoverMP software (Refeyn).

### Enzyme activity measurements

CA activity of CpNce103p was assessed using a stopped-flow spectrophotometer (SFM 3000, BioLogic) with 0.2 mM phenol red as a pH indicator, monitored at 557 nm. Reactions were performed in 20 mM HEPES–Na buffer (pH 7.5), containing 20 mM Na_2_SO_4_ to maintain ionic strength, using 3.4 mM CO_2_ as substrate. The enzyme (2.2 nM) and inhibitors were preincubated for 15 min at room temperature. Reactions were conducted at 25 °C and monitored for 30 s. Initial velocities were calculated from the linear portion of the absorbance curve (first 10–30 s) and corrected by subtracting the uncatalyzed CO_2_ hydration rate. All measurements were performed in quintuplicate and analyzed using GraphPad Prism.

### Crystallization

For crystallization, Δ42_CpNce103p was concentrated to 20 mg/mL in a buffer containing 50 mM Tris (pH 8.0) and 2 mM TCEP. Screening for crystal growth conditions was performed using the sitting-drop vapor diffusion technique in 96-well plates with commercial screens (PEGs Suite I, JCSG Core I Suite, and Morpheus; Molecular Dimensions). Sitting drops were set up in 96-well plates (Hampton Research) using a mosquito LCP robot (SPT Labtech) in a 1:1 protein-to-well solution ratio. Initial protein concentrations were 15 mg/mL and 7.5 mg/mL. Optimized crystals were obtained in 0.1 M sodium acetate (pH 5.0), 7.5 % (w/v) PEG 6000, and 20 % glycerol, with a final protein concentration of 7.5 mg/mL. The crystals were cryoprotected in mother liquor without additional cryoprotection and flash-frozen in liquid nitrogen.

### Structure determination

X-ray diffraction data for the first set of crystals were collected at 100 K on beamline 14.1 of the BESSY II synchrotron (operated by Helmholtz-Zentrum Berlin) using a wavelength of 0.9184 Å. The crystals diffracted to a resolution of 2.6 Å. Data integration and scaling were performed using XDS (Kabsch, 2010). The crystal structure was solved by molecular replacement using *S. cerevisiae* ScNCE103p (PDB ID: 3EYX) as the search model. The initial model was obtained with Phaser v2.8.3 (McCoy et al., 2007), followed by further refinement with phenix.refine (Afonine et al., 2018) from the Phenix package (v1.20.1-4487) and manual model building in Coot v0.9.8.7 (Emsley et al., 2010). Statistics for data collection, processing, structure solution, and refinement were calculated with phenix. and are summarized in Table 1. Structural figures were generated with PyMOL Molecular Graphics System v2.5.4 (Schrödinger, LLC).

**Table 1:** Data collection and refinement statistics.

Resolution range	41.05-2.701 (2.798-2.701)
Space group	P 41 21 2
Unit cell	118.788 118.788 188.214 90 90 90
Total reflections	70,338 (7379)
Unique reflections	35,198 (3693)
Multiplicity	2.0 (2.0)
Completeness (%)	93.11 (99.54)
Mean I/sigma(I)	15.96 (0.84)
Wilson B-factor	70.93
R-merge	0.03531 (0.7645)
R-meas	0.04994 (1.081)
R-pim	0.03531 (0.7645)
CC1/2	1 (0.712)
CC*	1 (0.912)
Reflections used in refinement	35,101 (3678)
Reflections used for R-free	1757 (185)
R-work	0.2723 (0.3909)
R-free	0.3288 (0.4011)
CC(work)	0.937 (0.767)
CC(free)	0.928 (0.681)
Number of nonhydrogen atoms	58,361
macromolecules	5769
ligands	80
solvent	33
Protein residues	815
RMS(bonds)	0.004
RMS(angles)	0.82
Ramachandran favored (%)	93.06
Ramachandran allowed (%)	6.57
Ramachandran outliers (%)	0.37
Rotamer outliers (%)	1.09
Clashscore	12.10
Average B-factor	90.91
macromolecules	91.00
ligands	84.46
solvent	74.51
Number of TLS groups	1

### Accession codes

Coordinates and structure factors for CpNce103p have been deposited in the Protein Data Bank (PDB) under accession code 9S4G (Δ42_CpNce103p).

### Molecular docking

Chain A of the crystal structure of Δ42_CpNce103p in complex with acetazolamide (this work; PDB ID: 9S4G) was used as the starting structure. Docking was performed using Molecular Operating Environment (MOE, Chemical Computing Group, 2022) and AutoDock 4.2.6 (Morris et al., 2009). In MOE, docking employed the default settings of the Dock module. In AutoDock, docking calculations were performed using the Zn^2+^-parametrized force field, which accounts for the coordination geometry and partial covalent interaction of the zinc ion with nearby ligand atoms (Santos-Martins et al., 2014). The docking grid was centred on the Zn^2+^-containing catalytic pocket, with default values used for the remaining protocol. Finally, docking poses were filtered based on binding energy and the spatial proximity of the sulfonamide nitrogen to the catalytic Zn^2+^ ion (<3 Å). To account for scoring variability across docking engines, we evaluated each compound using four distinct MOE scoring functions (London dG, Affinity dG (AdG), Alpha HB (AHB), and ASE scores) and the AutoDock scoring function. Incorporating multiple scoring schemes helps reduce bias from any single model and improves robustness in compound prioritization.

To integrate the results across these five methods, we employed an exponential consensus ranking (ECR) approach (Palacio-Rodriguez et al., 2019). ECR combines rankings from multiple scoring functions using an exponential distribution to weight each rank. For each molecule *i*, an exponential score *p(r_ij_)* is computed for each docking method *j* based on its rank *r_ij_*. The final consensus score is the sum of the exponential scores across all methods, prioritizing compounds consistently ranked highly, and computed as follows:

$$P\left(i\right)=\sum_{j}p\left(r_{i}^{j}\right)=\frac{1}{\sigma }\sum_{j}esp\left(-\frac{r_{i}^{j}}{\sigma }\right)$$

Equation 1: Exponential consensus ranking calculation where *r_ij_* is the rank of compound *i* in scoring function *j*, *n* is the total number of scoring functions, and *σ* is a scaling parameter controlling the steepness of the exponential decay, typically set to the expected rank value.

## Results and discussion

### Cloning, expression, and purification of truncated CpNce103p constructs

**Fig. 1:**
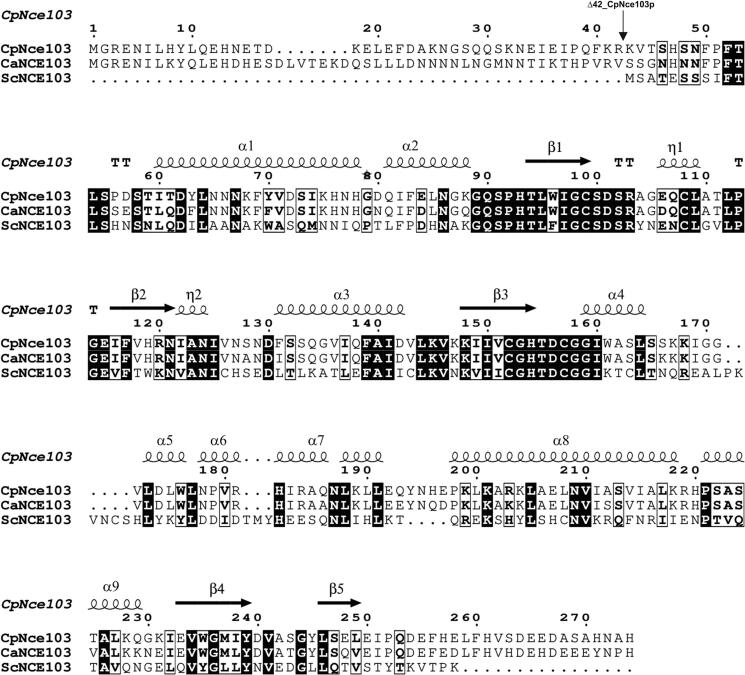
Multiple sequence alignment of CpNCE103 (*Candida parapsilosis,* G8B6R8), CaNCE103 (*Candida albicans*, Q5AJ71), and ScNCE103 (*Saccharomyces cerevisiae,*P53615). Secondary structure elements, derived from the crystal structure of CpNCE103, are shown above the sequences, with α-helices (α1–α9) represented as cylinders and β-strands (β1–β5) as arrows. Conserved residues are highlighted in black boxes, with full conservation indicated by white letters on a black background. The black arrows indicate sites of truncation. All sequences were obtained from NCBI databases.

To optimize CpNce103p for structural studies, two constructs were prepared: the full-length protein (residues 1–295) and an N-terminally truncated variant lacking the first 42 residues (Δ42; residues 43–274) (Fig. 1). Both constructs were cloned into a modified pET-22b(+) vector lacking the pelB leader sequence for periplasmic localization. The native stop codon was retained in each construct to avoid fusion with a C-terminal His-tag, relying instead on naturally occurring histidine residues at the C-terminus for Ni^2+^-affinity purification. Construct design was guided by multiple sequence alignments of fungal β-carbonic anhydrases and supported by AlphaFold structural predictions, which indicated that the N-terminal region up to approximately residue 40 is structurally disordered and likely to hinder crystallization. Both constructs were successfully expressed in *E. coli* and purified to near-homogeneity. However, only the Δ42 construct, which fully excludes the predicted disordered region and C-terminal tail, yielded well-diffracting crystals. The full-length protein did not crystallize under the tested conditions, underscoring the importance of truncating flexible terminal regions to facilitate crystal formation and improve structural stability.

To maximize the yield of both CpNce103 variants, we systematically optimized expression conditions, including temperature (20–37 °C), induction time (4–20 h), and IPTG concentration (0.2–1.0 mM). The highest yields of proteins were obtained by inducing expression with 0.4 mM IPTG and cultivating at 20 °C for 24 h post-induction. The presence of a reducing agent, such as TCEP, was essential to maintain protein solubility during purification. CpNce103p and Δ42_CpNce103p were successfully purified under these conditions. Typical yields ranged from 15 to 28 mg of purified protein per liter of culture for the full-length and Δ42 variants. Enzymatic activity was assessed using a stop-flow pH/dye indicator assay, and the results confirmed that the truncated construct retained catalytic activity comparable to the wild-type protein. Additionally, the presence of TCEP in the reaction buffer did not adversely affect enzyme activity, supporting its use during the purification.

### Tetrameric assembly of CpNce103 in solution

**Fig. 2:**
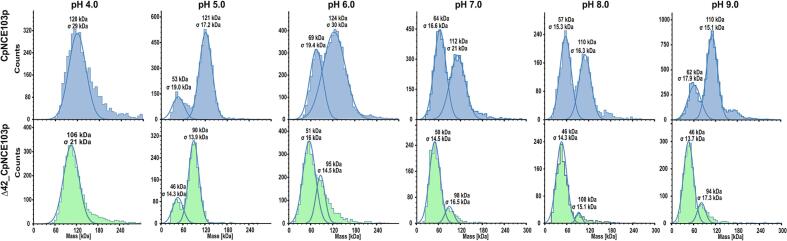
pH-dependent oligomerization of CpNce103p and Δ42_CpNce103p. Mass photometry histograms for full-length CpNce103p (top panels, blue) and the truncated variant Δ42_CpNce103p (bottom panels, green) were measured across a pH range of 4.0 to 9.0. Each panel displays the molecular mass distribution (kDa) and counts of detected particles, with fitted Gaussian curves indicating the presence of distinct oligomeric species. (For interpretation of the references to colour in this figure legend, the reader is referred to the web version of this article.)

The oligomeric state of CpNce103p and Δ42_CpNce103p over a pH range of 4.0 to 9.0 was analyzed by mass photometry (Fig. 2), which enables the precise determination of molecular mass distributions at nanomolar concentrations, offering insight into the solution behavior of proteins under near-physiological conditions. At acidic pH (4.0–5.0), full-length CpNce103p existed predominantly as a tetramer (∼123 kDa), consistent with its crystallographic quaternary structure. At pH 6.0, both dimeric (∼61 kDa) and tetrameric species were observed, suggesting an equilibrium between these states. This bimodal distribution persisted at pH 7.0–8.0, with the dimer becoming the dominant form and the tetramer remaining as a minor population. Interestingly, at pH 9.0, the tetramer again became more prominent, indicating a potential restabilization of the higher-order assembly under alkaline conditions.

In contrast, the Δ42_CpNce103p variant displayed markedly altered behavior. At pH 4.0, it retained partial tetramer formation (∼102 kDa), but at higher pH values the equilibrium shifted strongly toward lower-order species, with predominant dimers (∼51 kDa) and minor higher-order aggregates. Notably, the tetrameric form never became dominant in the truncated variant, highlighting the essential role of the N-terminal 42 residues in promoting and stabilizing tetramerization. This highlights the critical role of the N-terminal region in stabilizing higher-order oligomeric states – a function similarly attributed to the helical arms in CaNce103p and the N-terminal extension in ScNce103p (Dostal et al., 2018, Teng et al., 2009).

Together, these results indicate that CpNce103p assembles into a dynamic equilibrium of dimers and tetramers, modulated by environmental pH. The N-terminal extension plays a crucial role in mediating dimer–dimer interactions, supporting the formation of the functional tetrameric assembly observed in the crystal structure.

### Testing of inhibitors

**Fig. 3:**
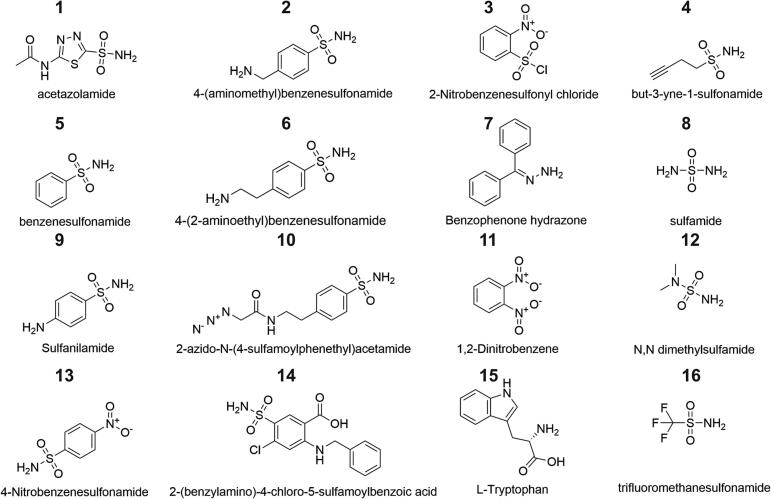
Structures of compounds (1–16) investigated as inhibitors of CpNce103p.

Inhibition constants (Ki) for 16 sulfonamide derivatives (R–SO_2_–NH_2_), sourced from the in-house compound library at the Institute of Organic Chemistry and Biochemistry, Prague, were measured using the full-length CA (structures shown in Fig. 3; inhibition data in Table 2). The Ki values spanned over three orders of magnitude, ranging from single-digit micromolar to millimolar values. Acetazolamide (compound 1), a clinically used standard CA inhibitor, inhibited CpNce103p with the most potency, achieving a Ki of 1.2 µM. Trifluoromethanesulfonamide (compound 16), which features a markedly inductive electron-withdrawing (−I) CF_3_ group, showed comparable potency (Ki = 2.2 µM), reinforcing the importance of low sulfonamide basicity for effective Zn^2+^ coordination in the active site (Pecina et al., 2013).

**Table 2:** Inhibition summary.

ID	Inhibitor	Ki (µM)
1	Acetazolamide	1.2
2	4-(aminomethyl)benzenesulfonamide	3166
3	2-Nitrobenzenesulfonyl chloride	869
4	but-3-yne-1-sulfonamide	933
5	Benzenesulfonamide	922
6	4-(2-aminoethyl)benzenesulfonamide	451
7	Benzophenone hydrazone	302
8	Sulfamide	1595
9	Sulfanilamide	825
10	2-azido-N-(4-sulfamoylphenethyl)acetamide	1515
11	1,2-Dinitrobenzene	153
12	N,N dimethylsulfamide	ND*
13	4-Nitrobenzenesulfonamide	188
14	2-(benzylamino)-4-chloro-5-sulfamoylbenzoic acid	377
15	L-Tryptophan	842
16	Trifluoromethanesulfonamide	2.2

ND* inhibition not detected.

Ortho- or para-nitro-substituted benzenesulfonamides, such as 4-nitrobenzenesulfonamide (compound 13, Ki = 188 µM) and 1,2-dinitrobenzenesulfonamide (compound 11, Ki = 153 µM), exhibited two orders of magnitude weaker inhibition, yet still performed better than unsubstituted benzenesulfonamide (compound 5, Ki = 922 µM) and sulfanilamide (compound 9, Ki = 825 µM). This trend is consistent with the resonance electron-withdrawing (–M) capacity of the nitro group compared with the resonance electron-donating (+M) capacity of the aniline group. Such substituent characteristics stabilize or destabilize, respectively, the deprotonated sulfonamide NH^−^ group, crucial for Zn^2+^ binding (Krishnamurthy et al., 2008). One of the least effective inhibitors was 2-azido-N-(4-sulfamoylphenethyl)acetamide (compound 10, Ki = 1515 µM), highlighting the detrimental effect of bulky substituents that hinder access to the Zn^2+^ site. In summary, the inhibitory potency of these compounds against fungal β-CAs is determined by their electronic properties as well as their steric accessibility.

### Enzyme structure of CpNce103p in complex with acetazolamide

**Fig. 4:**
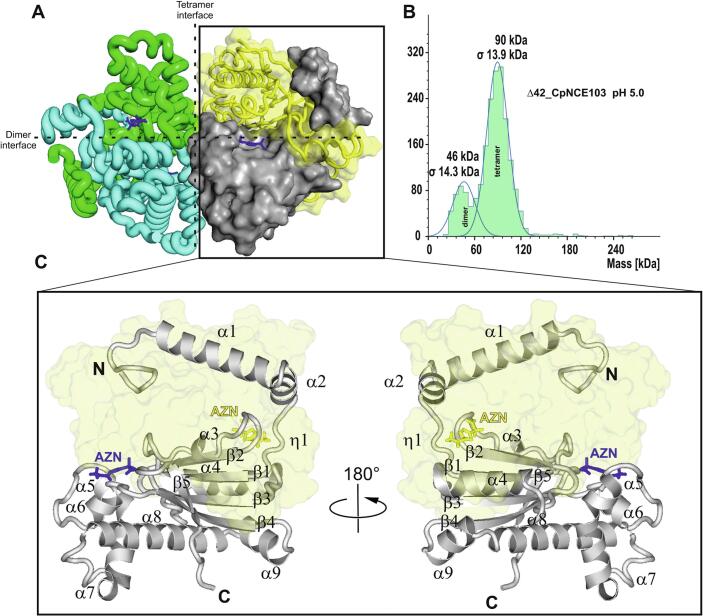
Oligomeric structure and active-site architecture of CpNce103p. (A) Tetrameric assembly of CpNce103p shown as a dimer of dimers. Two orthogonal dimerization interfaces are indicated. Subunits are colored green, cyan, yellow, and gray. The bound inhibitor acetazolamide (AZM) is shown in blue at the dimer interface. (B) Mass photometry analysis of CpNce103p at pH 5.0 reveals two main populations corresponding to the dimer and tetramer. (C) Structural view of the active site located at the dimer interface. Two opposing monomers are shown in cartoon and surface representation, including labeled α-helices and β-strands. The bound AZM (blue) coordinates the active-site zinc ion. A 180° rotated view of the complex is shown for clarity. Key secondary structural elements lining the substrate tunnel are highlighted. (For interpretation of the references to colour in this figure legend, the reader is referred to the web version of this article.)

The crystal structure of CpNce103p in complex with acetazolamide (AZM) reveals a homotetramer organized as a dimer of dimers (Fig. 4). Each dimer consists of two subunits tightly associated via extensive interfaces, and two such dimers further associate to form the tetramer. This quaternary arrangement is identical to that observed in the *Candida albicans* homolog CaNce103p (Dostal et al., 2018) and other “plant-type” β-carbonic anhydrases, where the dimerization and tetramerization interfaces are roughly perpendicular to each other. Substrate tunnels are located at each dimer interface, running between the two monomers. These tunnels lead toward the active sites and likely serve as the entry or exit pathways for the substrate (CO_2_/HCO_3_^−^) in the tetrameric enzyme. Consistent with this, the active site of each monomer is positioned near the dimer interface, with the neighboring subunit contributing certain active-site residues. Each monomer in the tetramer is bound to one AZM molecule (one inhibitor per active site), yielding a fully occupied tetramer with four bound inhibitors.

Each CpNce103p monomer adopts the typical β-CA fold and can be divided into three regions. First, an N-terminal α-helical region (approximately two helices) extends outward from the core and mediates dimerization. These N-terminal helices from two monomers pack against each other, stabilizing the dimer interface. In CaNce103p and ScNce103p, this N-terminal region is flexible: truncations were required for crystallization, underscoring its mobility, but the remaining helices still suffice for dimer formation. Second, a conserved central core of the enzyme comprises five β-strands and nine α-helices. The β-strands (β1–β5) form the backbone of the β-CA fold, and the α-helices (α3–α9) surround the β-sheet, shaping the globular enzyme and providing the scaffold for the active site. Finally, a C-terminal helical subdomain, a small helical segment near the C-terminus, lies at the periphery of the monomer and contributes to tetramerization by interfacing with the adjacent dimer. In the tetramer, these C-terminal helices from each monomer flank the neighboring protomers, creating additional contacts that further stabilize the dimer–dimer association. The C-terminus of CpNce103p is disordered in our structure: no density corresponding to an expected helix (often labeled “α12” in homologous structures) was observed. The absence of this helix in CpNce103p does not prevent tetramer formation, as other interactions suffice to hold the assembly together.

Analysis of the interfaces using PDBePISA confirmed that the tetramer is a stable biological assembly. The dimer interface buries a large surface area involving numerous hydrophobic contacts and hydrogen bonds provided by the N-terminal helices and adjacent loops, corroborating that each pair of monomers forms a strong dimer. The dimer-of-dimers interface (bringing two dimers together) is somewhat smaller but still significant, mediated in part by the C-terminal subdomain helix and surrounding regions from each monomer.

CpNce103p displays a high degree of similarity to its fungal homologs in both sequence and three-dimensional structure. In particular, CpNce103p shares 85.3 % amino acid sequence identity with *C. albicans* CaNce103p (PDB ID: 6GWU), and the two structures superimpose with an RMSD of 0.357 Å over 1167 aligned atoms. In contrast, the *A. fumigatus* β-CA CafA (PDB ID: 7COJ) is only 38.2 % identical in sequence to CpNce103p, aligning with a larger RMSD of 0.635 Å over 856 atoms. The *S. cerevisiae* enzyme ScNCE103p (PDB ID: 3EYX) shows an intermediate 42.2 % sequence identity to CpNce103p, corresponding to an RMSD of 0.811 Å over 902 aligned atoms. Despite the lower sequence identity of the latter two homologs, these structural alignments confirm that CpNce103p retains the conserved β-carbonic anhydrase fold, with only modest deviations in backbone conformation (RMSD <1 Å) relative to the more divergent fungal orthologs.

**Fig. 5:**
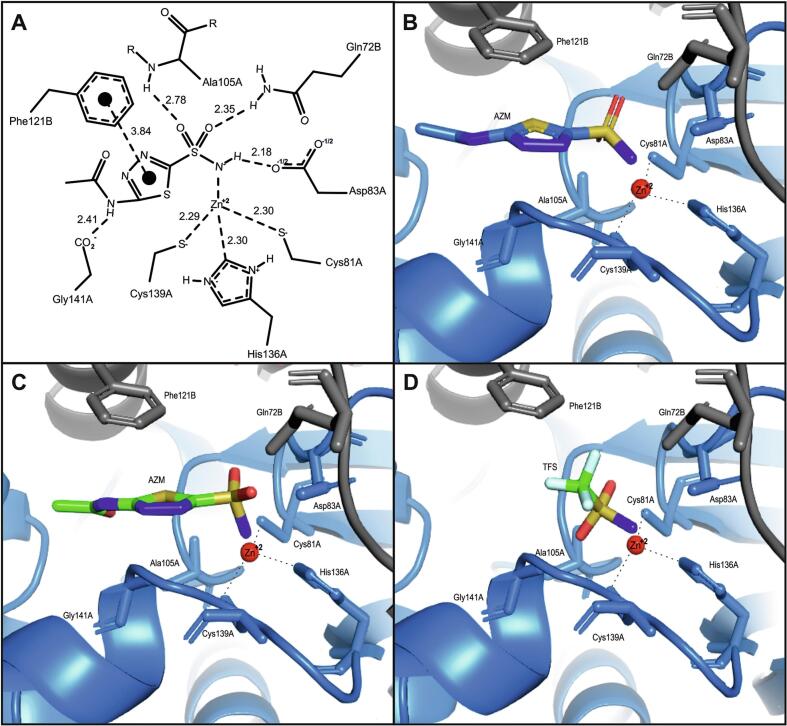
Structural interactions of acetazolamide (AZM) in the active site of CpNce103p. (A) 2D schematic diagram generated using PoseView, showing the binding mode of AZM. The Zn^2+^ ion is tetrahedrally coordinated by the side chains of Cys81A, His136A, Cys139A, and the sulfonamide nitrogen of AZM. Hydrogen bonds occur between AZM and residues Asp83A, Gln72B, Ala105A, and Gly141A. A π–π stacking interaction is formed with Phe121B from the adjacent subunit. Dashed lines represent metal coordination and hydrogen bonding. Distances are given in angstroms (Å). (B) 3D view of the AZM–CpNCE103 crystal structure rendered in PyMOL. AZM is shown in stick representation (blue carbon atoms chain A, black chain B), Zn^2+^ as a red sphere, and key interacting residues as labeled sticks. The protein backbone is depicted as a cartoon. (C) The minimum-RMSD pose (pose 4) from AutoDock shows docking of AZM in the CpNCE103 binding site. (D) Pose 8 of trifluoromethanesulfonamide (TFS) from AutoDock shows docking in the CpNCE103 binding site. (For interpretation of the references to colour in this figure legend, the reader is referred to the web version of this article.)

In the AZM-bound CpNce103p structure, Zn^2+^ is tetrahedrally coordinated by Cys81A, His136A, Cys139A, and the sulfonamide nitrogen of AZM (Fig. 5). This arrangement, typical of β-class carbonic anhydrases, features Zn–ligand distances around 2.3 Å. The sulfonamide group replaces the catalytic water as the fourth Zn^2+^ ligand. The O1 atom of the sulfonamide group as well as the O3 atom of the AZM acetamide group make chalcogen bonds with the sulfur of the thiadiazole ring (distances of 3.1 and 2.8 A, respectively), stabilizing its conformation (Kriz et al., 2018). The active site lies at the dimer interface, with residues from both subunits involved in AZM binding. Key contributions include His136A and Asp83A from chain A, and Gln72B and Phe121B from chain B. AZM forms several hydrogen bonds (Fig. 5). Its sulfonamide NH^−^ group donates an H-bond to Asp83A, while one sulfonyl oxygen accepts an H-bond from Gln72B. The other sulfonyl oxygen interacts with the backbone NH groups of Ala105A and Gly141A. These contacts stabilize the inhibitor in the pocket. Hydrophobic and aromatic interactions further reinforce binding. The thiadiazole ring of AZM engages in a π–π stacking interaction with Phe121B from the adjacent subunit (∼3.8 Å), while surrounding nonpolar residues form additional van der Waals contacts.

### Superposition and sequence comparison of fungal β-CA homologs

**Fig. 6:**
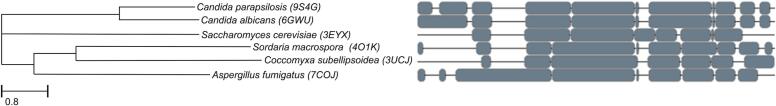
Phylogenetic tree, with relationships and multiple alignment shown as blocks of selected fungal β-carbonic anhydrases (NCE103 homologs).

We first focused on the three fungal β-carbonic anhydrases for which inhibition data were experimentally determined: CpNce103p (*Candida parapsilosis*, **G8B6R8**, PDB ID: 9S4G), CaNce103p (*Candida albicans*, Q5AJ71, PDB ID: 6GWU) (Dostal et al., 2018), and ScNce103 (*Saccharomyces cerevisiae*, P53615, PDB ID: 3EYX) (Teng et al., 2009). To place these enzymes in a broader structural context, we surveyed the Protein Data Bank (Burley et al., 2023) to identify additional well-characterized fungal β-CAs. Based on sequence and structural availability, we selected β-CAs from *Coccomyxa* sp. PA (3UCJ) (Huang et al., 2011); *Sordaria macrospora* (4O1K) (Lehneck, 2014), and *Aspergillus fumigatus* (7COJ) (Kim et al., 2020) for comparative analysis (Fig. 6).

Structural superpositions revealed a high degree of similarity between CpNce103p and CaNce103p, with a low backbone RMSD of 0.534 Å, consistent with their close evolutionary relationship and conserved catalytic motifs. Alignment with ScNCE103 from *Saccharomyces cerevisiae* yielded an RMSD of 1.42 Å, indicating a similar overall fold. Comparisons with *Coccomyxa* β-CA (3UCJ) and *Sordaria macrospora* β-CA (4O1K) showed RMSD values of 0.89 Å and 1.32 Å, respectively. In contrast, alignment with the *Aspergillus fumigatus* structure (7COJ) exhibited a substantially higher RMSD (13.56 Å), partly influenced by an N-terminal extension of approximately 75 amino acids, which appears structurally disordered or unrelated to the conserved β-CA fold.

Overall, these structural comparisons confirmed that CpNce103p shares a high degree of similarity with its fungal homologs, maintaining a conserved fold and active-site geometry. The larger structural deviation from CafA (*A. fumigatus*) was attributed to a unique N-terminal extension absent in CpNce103p.

### Molecular docking

Docking of trifluoromethanesulfonamide (Table 2, inhibitor 16) into the active site of CpNce103p β-carbonic anhydrase was conducted to estimate its binding mode. To validate the method, AZM was first redocked into the CpNce103p crystal structure determined in this work (PDB ID: 9S4G). Nine AutoDock poses of AZM gave RMSD values relative to the crystal pose of AZM ranging from 1.27 to 6.59 Å (Table 3). The minimum-RMSD pose (pose 4) from AutoDock exhibited a very similar binding mode to that of the crystal, displaying classical coordination of the sulfonamide nitrogen to the Zn^2+^ ion and hydrogen bonding to key active-site residues (Fig. 5B, C). The poses of trifluoromethanesulfonamide (Table 2, inhibitor 16) were more variable: pose 8 from AutoDock adopted a binding mode closely resembling that of AZM (Fig. 5D).

**Table 3:** Root-mean-square deviation (RMSD) values (Å) of docking poses relative to the CpNCE103 crystal structure, calculated without hydrogen atoms.

Pose	RMSD no H (Å)
Pose 1	3.14
Pose 2	6.59
Pose 3	2.72
Pose 4	1.27
Pose 5	4.70
Pose 6	5.81
Pose 7	4.72
Pose 8	5.57
Pose 9	4.90

In summary, CpNce103p shares fundamental structural features with other fungal β-CAs – a tetrameric assembly, the Cys–His–Cys zinc site, and a funnel-shaped active-site channel – while differing mainly in peripheral regions. The inhibition data confirm that CpNce103p binds sulfonamides via the conserved Zn^2+^ anchor and that its narrow active-site tunnel imposes stringent steric requirements. These findings provide a firm structural and functional basis for the rational design of CpNce103p-targeted inhibitors.

## CRediT authorship contribution statement

**Jiří Dostál:** Writing – original draft, Methodology, Data curation. **Zdeňka Uhrová:** Methodology. **Magdalena Škrlová:** Methodology. **Stanislav Macháček:** Methodology, Data curation. **Kamila Clarová:** Writing – original draft, Methodology, Investigation, Data curation. **Martin Lepšík:** Writing – original draft, Validation, Methodology, Investigation, Data curation. **Ondřej Bulvas:** Methodology, Investigation. **Milan Vrábel:** Supervision. **Olga Heidingsfeld:** Writing – original draft, Methodology, Conceptualization. **Iva Pichová:** Writing – original draft, Funding acquisition, Conceptualization.

## Declaration of competing interest

The authors declare that they have no known competing financial interests or personal relationships that could have appeared to influence the work reported in this paper.

## Data Availability

Data will be made available on request.
