# Supplementary material for: Structural characterization and inhibition of carbonic anhydrase from Candida parapsilosis
Source: J Struct Biol X. 2025 Nov 12;12:100140. doi: 10.1016/j.yjsbx.2025.100140 (PMC12664589; doi:10.1016/j.yjsbx.2025.100140)
Supplement: Supplementary Data 1 [file mmc1.docx]

**Structural characterization and inhibition of carbonic anhydrase from *Candida parapsilosis***

Jiří Dostál^1^, Zdeňka Uhrová^1^, Magdalena Škrlová^1^, Stanislav Macháček^1^, Kamila Clarová^1^, Martin Lepšík^1^, Ondřej Bulvas^1^, Milan Vrábel^1^, Olga Heidingsfeld^1,2^, and Iva Pichová^1^

^1^Institute of Organic Chemistry and Biochemistry of the Czech Academy of Sciences, Flemingovo náměstí 2, 166 10 Prague, Czech Republic

^2^Department of Biochemistry, Faculty of Science, Charles University in Prague, Hlavova 2030, 128 43 Prague, Czech Republic

**Correspondence:**

Iva Pichová, iva.pichova@uochb.cas.cz

# Supplementary material

**Table S1**. Primers used in the study

| **Construct** | **Primer name** | **Sequence** |
| --- | --- | --- |
| CpNce103p | WT_CP-FOR | ATTCGGATCCGAATTCGGGTAGAGAAAACATCTTAC |
|  | WT/∆26/∆42-REV | GGTGGTGGTGCTCGACTAATGTGCATTATGAGCT |
| ∆42_CpNce103p | ∆42_CP-FOR | ATTCGGATCCGAATTGTAAAGTCACTAGCCATTC |
|  | WT/∆26/∆42-REV | GGTGGTGGTGCTCGACTAATGTGCATTATGAGCT |
